# MiR-146a and miR-196a-2 polymorphisms are associated with hepatitis virus-related hepatocellular cancer risk: a meta-analysis

**DOI:** 10.18632/aging.101160

**Published:** 2017-01-31

**Authors:** Tian Tian, Meng Wang, Wenge Zhu, Zhi-Ming Dai, Shuai Lin, Peng-Tao Yang, Xing-Han Liu, Kang Liu, Yu-Yao Zhu, Yi Zheng, Meng Liu, Zhi-Jun Dai

**Affiliations:** ^1^ Department of Oncology, Second Affiliated Hospital of Xi'an Jiaotong University, Xi'an 710004, China; ^2^ Department of Biochemistry and Molecular Medicine, The George Washington University Medical School, Washington, DC 20037, USA; ^3^ Department of Anesthesia, Second Affiliated Hospital of Xi'an Jiaotong University, Xi'an 710004, China

**Keywords:** hepatitis virus-related hepatocellular carcinoma, microRNA, single nucleotide polymorphism, meta-analysis

## Abstract

Previous studies have investigated the role of miR-146a rs2910164 and miR-196a-2 rs11614913 polymorphisms in hepatocellular carcinoma (HCC) susceptibility, but the results are contradictory and few specifically studied hepatitis virus-related HCC. Therefore, we conducted a meta-analysis to evaluate the association between these two polymorphisms and hepatitis virus-related HCC risk. We performed a systematical search in EMBASE, PubMed, Web of Science, CNKI and Wanfang databases as of 25th November, 2016. Finally, we assessed 14 studies involving 3852 cases and 5275 controls. Our results suggest that rs2910164 has a significant association with increased hepatitis virus-related HCC risk in allelic, homozygous, heterozygous, and dominant models (CG+GG vs. CC: OR=1.22, 95% CI=1.06-1.39, P=0.004), particularly in Chinese and HBV-related HCC subgroups. Conversely, rs11614913 was associated with lower hepatitis virus-related HCC risk in the overall analysis under allelic (T vs. C: OR=0.85, 95% CI=0.74-0.98, P=0.02), homozygous, dominant and recessive models. Subgroup analyses showed decreased risk in Chinese, HBV- and HCV-related HCC. In conclusion, miR-146a C>G (rs2910164) can increase HBV-related HCC risk while miR-196a-2 C>T (rs11614913) may decrease the risk of HBV- and HCV-related HCC, especially in the Chinese population. Further, large-scale studies including other races are required to confirm these findings.

## INTRODUCTION

Hepatocellular carcinoma (HCC) is one of the leading causes of cancer death worldwide, especially in less-developed countries. [1]. It is well known that the main environmental risk factor of HCC is hepatitis virus infection. Approximately 80% cases of HCC are attributed to chronic infection by hepatitis B (HBV) or C virus (HCV). However, only a small number of infected people develop HCC in their lifetime, indicating the pivotal role of genetic factors in tumorigenesis [2, 3].

MicroRNAs (miRNA) are a class of small non-coding RNAs containing approximately 22 nucleotides. These endogenous molecules regulate gene expression via post-transcriptional repression. This regulation modulates diverse biological processes including proliferation, differentiation, and apoptosis, which are key steps in tumorigenesis [4, 5]. MiRNAs have been implicated in the pathogenesis of various forms of liver disease such as viral hepatitis, liver fibrosis, and cancer [6]. They can also serve as repressors of viral infection pathways, thus regulating the host-virus interaction [7]. Moreover, studies have revealed that miRNAs can act as tumor suppressors or oncogenes and participate in the HBV-related HCC development [8, 9]. Of all the miRNAs, miR-146a and miR-196a-2 are the most commonly studied in HCC. The miR-146a gene resides on chromosome 5q33.3 and the miR-196a-2 gene is located in a region between homeobox (HOX) clusters HOXC10 and HOXC9 on chromosome 12. The expression of mature miR-146a is decreased while miR-196a-2 are over-expressed in hepatocellular carcinoma tissues, suggesting both of them play roles in the development of HCC [10, 11]. Single nucleotide polymorphism (SNP) is the most common form of mutation in the genome. Any minute alteration in miRNAs can be amplified by the numerous target genes, thus considerably increasing the possibility of carcinogenesis. Hence, genetic variation of miRNAs may alter the maturation, expression or lead to dysfunction of these molecules and potentially contribute to hepatitis virus-related HCC risk [4, 12, 13]. A number of studies have shown that several SNPs in miRNAs including miR-146a C>G (rs2910164) and miR-196a-2 C>T (rs2910164) are linked to the predisposition for HCC [10, 11, 14-29].However, the results of these studies are inconsistent. In this study, we systematically analyzed all relevant studies to assess the association of two common polymorphisms (rs2910164 and rs11614913) with hepatitis virus-related HCC risk.

## RESULTS

### Characteristics of included studies

A total of 392 potentially relevant studies were identified via a database search. Finally, 14 studies comprising 3852 hepatitis virus-related HCC patients and 5275 cancer-free controls were included. And Table 1 lists the characteristics of these studies. Of the 14 studies, 8 studies including 1717 cases and 2589 controls evaluated the relationship between rs2910164 and hepatitis virus-related HCC risk [10, 15, 16, 19, 22-25], while 3 included both HBV-related and HCV-related HCC. Nine studies including 3217 cases and 4163 controls evaluated the association of rs11614913 with hepatitis virus-related HCC risk [10, 11, 14, 17-21, 24], and 5 included both HBV and HCV-related HCC. All studies were conducted in China except 2 Turkish studies and 1 Korean study. Additionally, 12 studies were based on hospital data and 3 included the general population. Distributions of genotype in controls conformed to HWE in all the studies except for 2 [10, 20]. The detailed data of the allele and genotype distributions as well as HWE from each study are shown in Table S1.

**Table 1:** Characteristics of the studies included in the meta-analysis

First author	Year	Country	Ethnicity	Genotyping method	Source of control	Number of case/control	NOS score	SNP
Yan	2015	China	Asian	PCR-RFLP	HB	227/287	6	2
Zhou	2014	China	Asian	PCR-RFLP	HB	184/281	7	1,2
Kou	2014	China	Asian	PCR-RFLP	HB	208/532	6	2
Cong	2014	China	Asian	PCR-RFLP	HB	104/218	6	1
Hao	2013	China	Asian	PCR-RFLP	HB	169/282	7	2
Zhang	2013	China	Asian	MassARRAY	PB	771/998	7	1,2
Shan	2013	China	Asian	PCR-RFLP	HB	71/185	8	1
Han	2013	China	Asian	FQ-PCR	HB	1017/1009	7	2
Xiang	2012	China	Asian	PCR-RFLP	HB	73/100	6	1
Kim	2012	Korea	Asian	PCR-RFLP	PB	127/201	6	1,2
Wang	2011	China	Asian	MassARRAY	HB	199/384	8	1
Akkız	2011	Turkey	Caucasian	PCR-RFLP	HB	188/222	9	1
Akkız	2011	Turkey	Caucasian	PCR-RFLP	HB	153/185	9	2
Qi	2010	China	Asian	PCR-LDR	HB	361/391	8	2

PCR: polymerase chain reaction; RFLP: restriction fragment length polymorphism; FQ: fluorescent-probe real-time quantitative; LDR: ligation detection reaction; PB: population based; HB: hospital based; NOS: Newcastle-Ottawa Quality Assessment Scale; SNP: single-nucleotide polymorphism; SNP No.1: rs2910164, No 2: rs1161491.

### Meta-analysis results

**Figure 1:**
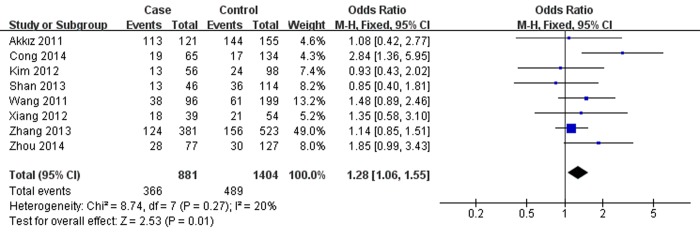
Forest plot describing the association between miR-146a rs2910164 and hepatitis virus-related HCC risk under homozygous model (GG *vs*. CC). The squares and horizontal lines correspond to the study-specific OR and 95% CI. The area of the squares reflects the weight (inverse of the variance). The diamond represents the summary OR and 95% CI. M–H: Mantel–Haenszel; df: degrees of freedom; Events: the number of GG genotypes.

As presented in Table 2, miR-146a rs291016 was found to increase hepatitis virus-related HCC risk in overall analysis under G *versus* (*vs*.) C (OR=1.13, 95% CI =1.04-1.24, *P*=0.006,), GG *vs*. CC (OR=1.28, 95% CI=1.06-1.55, *P*=0.01, Fig. 1), CG *vs*. GG (OR=1.20, 95% CI=1.04-1.38, *P*=0.01) and CG+GG *vs*. CC (OR=1.22, 95% CI=1.06-1.39, *P*=0.004). Additionally, this risk is more significant in the Chinese population. After omitting the study which did not conform to HWE, the results were found to be consistent with the overall analysis. Additionally, subgroup analyses showed a higher HBV-related HCC risk in the same 4 genetic models, whereas no significance was found in the HCV-related HCC subgroup. When stratifying data by source of control, rs2910164 was also observed to significantly increase hepatitis virus-related HCC risk based on hospital data, but there was no statistical significance based on population data.

**Table 2:** Meta-analysis results of miR-146a and miR-196a-2 polymorphisms with hepatitis virus-related HCC risk

SNP	B *vs*. A				BB *vs*. AA				AB *vs*. AA				BB *vs*. AA+AB				AB+BB *vs*. AA			
OR (95%CI)	*P*	I^2^	*Ph*	OR (95%CI)	*P*	I^2^	*Ph*	OR (95%CI)	*P*	I^2^	*Ph*	OR (95%CI)	*P*	I^2^	*Ph*	OR (95%CI)	*P*	I^2^	*Ph*
miR-146a C>G (rs2910164)																				
Overall	1.13 (1.04-1.24)	**0.006**	36%	0.14	1.28 (1.06-1.55)	**0.01**	20%	0.27	1.20 (1.04-1.38)	**0.01**	0%	0.95	1.10 (0.93-1.29)	0.26	35%	0.15	1.22 (1.06-1.39)	**0.004**	0%	0.81
HWE	1.12 (1.02-1.24)	**0.02**	44%	0.10	1.23(1.01-1.51)	**0.04**	18%	0.29	1.18 (1.01-1.37)	**0.04**	0%	0.95	1.07(0.90-1.26)	0.47	36%	0.15	1.19 (1.03-1.37)	**0.02**	0%	0.82
Chinese	1.17 (1.06-1.29)	**0.002**	39%	0.15	1.32 (1.08-1.62)	**0.007**	36%	0.16	1.19 (1.03-1.39)	**0.02**	0%	0.84	1.19 (0.99-1.43)	0.06	32%	0.20	1.23 (1.06-1.41)	**0.005**	0%	0.61
HB	1.20 (1.05-1.36)	**0.006**	47%	0.09	1.51 (1.14-1.99)	**0.004**	17%	0.31	1.23 (0.99-1.52)	0.06	0%	0.84	1.18 (0.94-1.46)	0.15	48%	0.09	1.29 (1.06-1.58)	**0.01**	0%	0.69
PB	1.08 (0.95-1.22)	0.25	0%	0.76	1.11 (0.85-1.45)	0.45	0%	0.64	1.17 (0.97-1.42)	0.10	0%	0.97	1.01 (0.79-1.29)	0.94	0%	0.59	1.16 (0.97-1.39)	0.11	0%	0.93
HBV-	1.13 (1.03-1.23)	**0.01**	39%	0.12	1.26 (1.04-1.54)	**0.02**	10%	0.36	1.20 (1.04-1.39)	**0.01**	0%	0.95	1.07 (0.91-1.27)	0.41	33%	0.16	1.21 (1.06-1.39)	**0.005**	0%	0.81
HCV-	1.25 (0.89-1.76)	0.20	0%	0.42	1.98 (0.89-4.45)	0.10	0%	0.39	1.18 (0.62-2.23)	0.62	0%	0.92	1.67 (0.75-3.71)	0.21	51%	0.13	1.38 (0.76-2.52)	0.30	0%	0.97
miR-196a2 C>T (rs11614913)																				
Overall	0.85 (0.74-0.98)	**0.02**	73%	0.00	0.71 (0.52-0.95)	**0.02**	75%	0.00	0.84 (0.70-1.01)	0.07	57%	0.02	0.82 (0.68-0.98)	**0.03**	54%	0.03	0.80 (0.65-0.99)	**0.04**	71%	0.00
HWE	0.88 (0.76-1.01)	0.07	72%	0.00	0.75 (0.55-1.02)	0.07	74%	0.00	0.87 (0.72-1.06)	0.16	55%	0.03	0.85 (0.71-1.02)	0.08	50%	0.05	0.83 (0.67-1.04)	0.11	69%	0.00
Chinese	0.85 (0.73-0.99)	**0.04**	76%	0.00	0.70 (0.50-0.97)	**0.03**	78%	0.00	0.82(0.67-1.02)	0.07	64%	0.01	0.83 (0.68-1.00)	0.05	56%	0.04	0.79 (0.62-1.00)	0.05	75%	0.00
HB	0.83 (0.69-0.99)	**0.04**	78%	0.00	0.66 (0.45-0.97)	**0.03**	79%	0.00	0.83 (0.67-1.04)	0.11	60%	0.02	0.77 (0.60-0.99)	**0.04**	63%	0.01	0.78 (0.60-1.01)	0.06	74%	0.00
PB	0.92 (0.70-1.22)	0.57	65%	0.09	0.85 (0.46-1.58)	0.60	68%	0.08	0.88 (0.53-1.45)	0.62	63%	0.10	0.86 (0.71-1.04)	0.13	0%	0.34	0.88 (0.51-1.52)	0.64	71%	0.06
HBV-	0.85 (0.73-0.98)	**0.03**	75%	0.00	0.70 (0.50-0.96)	**0.03**	77%	0.00	0.83 (0.69-1.01)	0.06	56%	0.02	0.82 (0.67-1.00)	0.05	58%	0.01	0.79 (0.63--0.99)	**0.04**	71%	0.00
HCV-	0.80 (0.65-0.98)	**0.03**	0%	0.92	0.61 (0.39-0.94)	**0.02**	0%	0.93	0.82 (0.59-1.12)	0.21	0%	0.69	0.69 (0.47-1.02)	0.06	0%	1.00	0.75 (0.55-1.02)	0.07	0%	0.72

PB: population based; HB: hospital based; HWE: subgroup excluding the studies departing from HWE; HBV-: hepatitis B virus-related HCC; HCV-: hepatitis C virus-related HCC; N: number of samples; A: the major allele; B: the minor allele; OR: odds ratio; CI: confidence interval; *Ph*: P-value of Q-test for heterogeneity.

**Figure 2:**
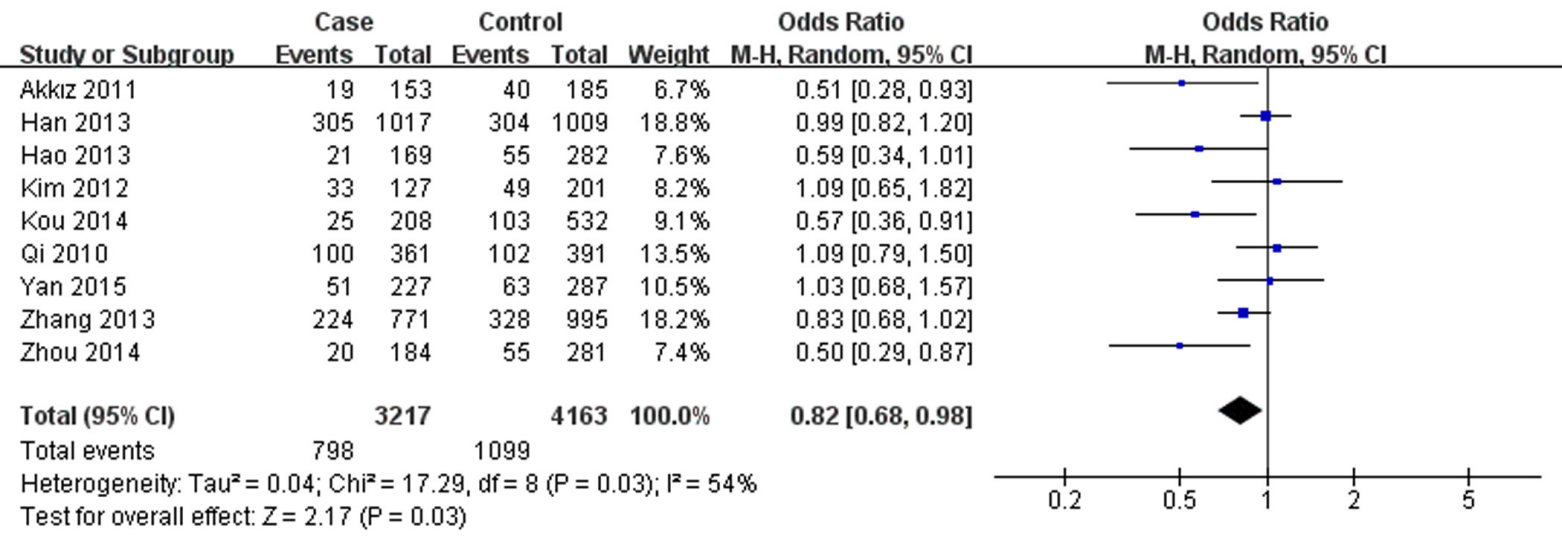
Forest plot describing the association between miR-196a-2 rs11614913 and hepatitis virus-related HCC risk under recessive model (TT *vs*. CC+CT). The squares and horizontal lines correspond to the study-specific OR and 95% CI. The area of the squares reflects the weight (inverse of the variance). The diamond represents the summary OR and 95% CI. M–H: Mantel–Haenszel; df: degrees of freedom; Events: the number of TT genotypes.

Conversely, miR-196a2 rs11614913 was associated with lower hepatitis virus-related HCC risk in overall analysis under T *vs*. C (OR=0.85, 95% CI=0.74- 0.98, *P*=0.02), TT *vs*. CC (OR=0.71, 95% CI=0.52-0.95, *P*=0.02), TT *vs*. CT+CC (OR=0.82 , 95% CI=0.68-0.98, *P*=0.03, Fig. 2) and CT+TT *vs*. CC (OR=0.80, 95% CI =0.65-0.99, *P*=0.04). The results were similar in the overall population as well as particularly among the Chinese. However, further analysis of the studies which were in agreement with HWE showed no associations between rs11614913 and hepatitis virus-related HCC. Additionally, in subgroup analysis by source of control, decreased hepatitis virus-related HCC risk was observed based on hospital data, however, no significance was shown based on population data. Furthermore, in the stratified analysis according to etiology, rs11614913 was found to decrease both HBV- and HCV-related HCC risk in different genetic models.

### Heterogeneity analysis

No significant heterogeneity was observed for rs2910164 in all the genetic models analyzed. However, significant heterogeneity was detected for rs11614913 in each genetic model (Table 2). To explore the source of heterogeneity, we carried out subgroup analysis. After stratifying by etiology, the value of I^2^ in HCV-related HCC subgroup was reduced to zero. However, heterogeneity was still significant in other subgroups. We then used Galbraith plots to further investigate the heterogeneity and identified 3 three studies [10, 17, 20] as the outliers (Fig. S1). When these 3 studies were excluded, I^2^ values were <50% in heterozygous and recessive models in the overall populations and some subgroups (data not shown).

### Sensitivity analysis and publication bias

**Figure 3:**
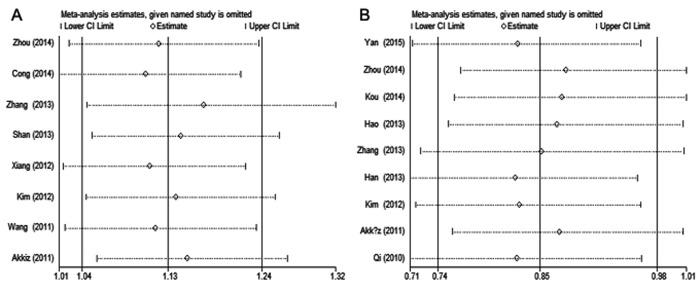
Sensitivity analysis of hepatitis virus-related HCC risk associated with (**A**) miR-146a rs2910164 and (**B**) miR-196a-2 rs11614913 under the allelic model. Pooled ORs were computed by omitting each study (left column) in turn. The two ends of the dotted lines represent the 95% CI.

**Figure 4:**
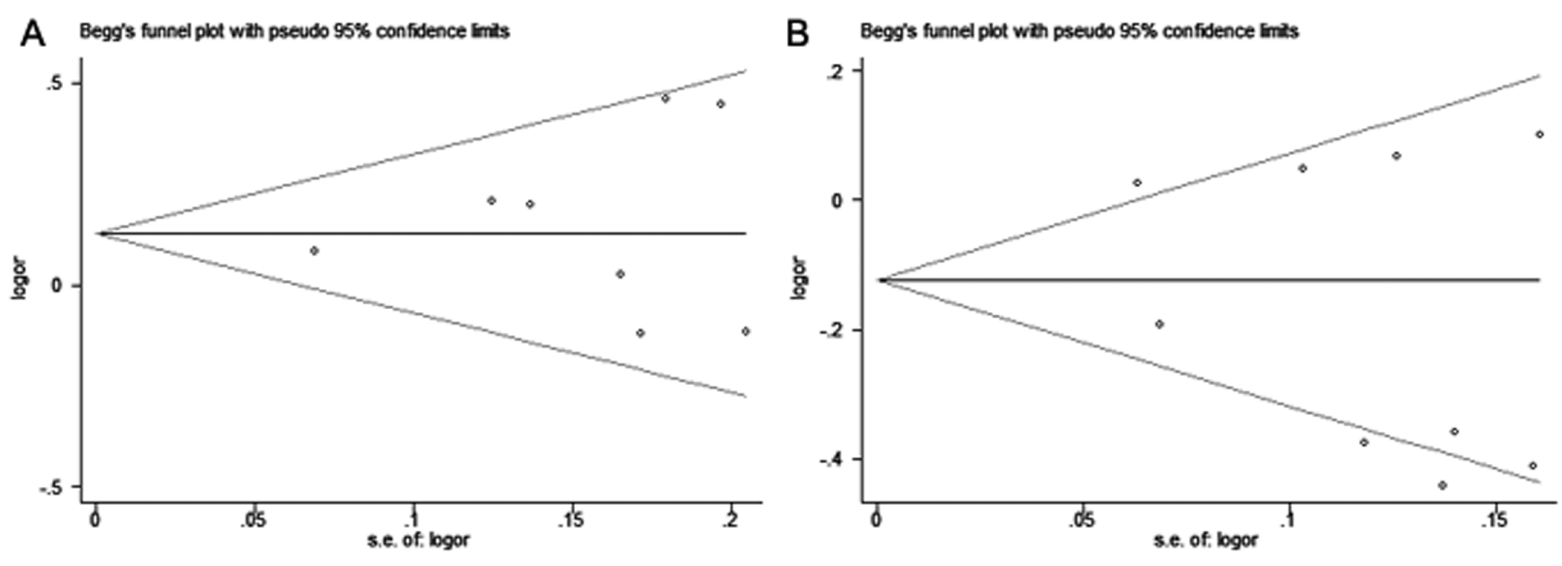
Begg's funnel plots for publication bias of (**A**) miR-146a rs2910164 and (**B**) miR-196a-2 rs11614913 polymorphisms with hepatitis virus-related HCC risk under allelic model. Each point represents a single study for the indicated association.

The result of sensitivity analyses shows that the omission of any individual study did not substantially alter the pooled ORs, suggesting that the meta-analysis results are robust and statistically reliable (Fig. 3). Begg's funnel plots did not show any obvious asymmetry in the overall analysis (Fig. 4). The Egger's test results were not significant either (Table S1). Thus, no publication bias was revealed for the 2 polymorphisms and hepatitis virus-related HCC risk.

## DISCUSSION

In recent years, several studies have focused on the SNPs in miRNAs and have investigated their potential role in HCC susceptibility [10, 11, 14-28]. A deeper understanding of the association between these polymorphisms and HCC would help early detection and prevention of this deadly disease in high-risk populations such as the Chinese. Amongst these SNPs, miR-146a C>G and miR-196a-2 C>T are the most commonly studied. Some case-control studies suggested these two SNPs contribute to HCC development [10, 11, 14, 16, 18, 20, 21, 24, 25], whereas other investigations failed to find any relationship between them [15, 17, 19, 22, 23]. The inconsistent results may originate from several factors including sample size, source of controls and genotype methods. Several meta-analyses have also explored the association between these 2 SNPs and HCC risk, but the results are inconsistent, while only few have focused specifically on hepatitis virus-related HCC [29-36]. Hence, we performed this updated meta-analysis to improve our understanding of the relationship between miR-146a C>G/miR-196a-2 C>T and hepatitis virus-related HCC risk, hoping to provide guidance for future studies.

Our results suggest that the G allele of rs2910164 increases the risk of hepatitis virus-related HCC, specifically for HBV-related HCC. This means that individuals carrying the G allele may be more susceptible to HBV-related HCC as compared to those carrying the C allele. Recent evidences indicated that miR-146a can promote apoptosis by inhibiting the NF-κB pathway and blocking its impact on cell proliferation, angiogenesis, metastasis and cancer cell survival. Loss of function of miR-146a could promote cancer cell migration and invasion[21]. And it has been reported that the rs2910164 polymorphism may reduce the production of mature miR-146a and lead to decreased suppression of its target genes including vascular endothelial growth factor, nuclear factor-κB, p65, and HAb18G, which are probably involved in hepatocarcinogenesis. Additionally, miR-146a expres-sion in hepatoma cells and tissues is much lower than that in normal hepatic cells and tissues [37, 38]. The results from the present meta-analysis confirmed the role of miR-146a rs2910164 in HCC development. Our results are consistent with that from previous meta-analyses [32-35], However, few differences were noted in our results compared with previous studies, since we specifically focused on hepatitis virus-related HCC. Although the *P*-value of HWE in the controls was <0.05 in one study [10], we included this study in the analysis since excluding it did not significantly influence the pooled OR. Additionally, neither significant heterogeneity nor publication bias was detected for this SNP, suggesting that our result regarding miR-146a C>G was statistically reliable.

Previous studies have revealed that miR-196a-2 deregulates numerous target genes such as HOX and ANXA1, which are considered to play crucial roles in the progression of HCC. Therefore, variations in miR-196a-2 may relate to HCC susceptibility [30, 39]. Our data support this role of miR-196a-2 in HCC development. However, our results should be interpreted cautiously, due to obvious heterogeneity existed. Subgroup analysis suggested that the major source of heterogeneity may be the type of hepatitis virus, while Galbraith plots identified the studies conducted by Kou, Zhou and Han as the outliers. For this SNP, one study deviated from HWE [20]. After excluding this study, no significant association was identified in any model. This indicates that this study did not investigate a broad representative population, which has greatly impacted the pooled ORs. Therefore, epidemiological studies with broader population are needed to confirm our findings. The conclusions of previous meta-analyses for this SNP and HCC susceptibility were contradictory. Some studies reported no association between rs11614913 and HCC risk [29, 30, 33] , whereas 2 recent meta-analyses published by Chen and Zhu showed that the C allele of rs11614913 may contribute to HCC susceptibility [35, 36]. Our results are consistent with those reported by Chen and Zhu. Nevertheless, our meta-analysis differs from these studies. Chen et al. assessed HCC risk without stratifying by etiology while we focused on hepatitis virus-related HCC. Though Zhu et al. specifically analyzed HBV-related HCC, there were only 7 studies in their meta-analysis. On the other hand, our analysis included 2 additional studies and we estimated both HBV- and HCV-related HCC risk.

We attempted a comprehensive evaluation of the relationship between miR-146a/miR-196a-2 poly-morphisms and hepatitis virus-related HCC risk. However, few limitations remain. First, the total sample size in this study was relatively small, especially for the HCV-related HCC subgroup. Additionally, we only examined the Chinese subpopulation as only one Korean and one Turkish study were included for both SNPs. Therefore, large-scale studies including multiple ethnicities are needed to provide sufficient statistical power. Secondly, heterogeneity still existed for rs11614913 in spite of our effort. And there may be a bias considering the representativeness of the samples. Therefore, it needs to be acknowledged that the result of this SNP, which may be affected, should be interpreted with caution. Thirdly, language bias may exist since only 2 languages were employed in the literature review. Lastly, lack of available original data prevented adjustment for other covariates such as age, gender, and lifestyle. As gene-environment interactions also affect cancer risk, these factors may potentially influence the results.

In summary, the results of our meta-analysis suggest that both miR-146a C>G and miR-196a-2 C>T polymorphisms associate with hepatitis virus-related HCC risk, especially in the Chinese population. MiR-146a C>G can increase HBV-related HCC risk while miR-196a-2 C>T may decrease the risk of both HBV-related and HCV-related HCC. In addition, large-scale studies with multi-ethnic groups are needed to confirm the findings. Detailed information about the effects of gene-environment interaction on HCC development is also required to further clarify these associations.

## MATERIALS AND METHODS

### Search strategy

All relevant literature, in English or Chinese, was identified from databases including EMBASE, PubMed, Web of Science, Wanfang and Chinese National Knowledge Infrastructure (CNKI) as of 25th November, 2016. The search was performed using the following terms: “miR-146a OR microRNA-146a OR miR-196a-2 OR microRNA-196a-2” AND “polymorphisms OR variant OR SNP OR mutation” AND “hepatocellular carcinoma OR liver cell carcinoma OR liver cancer”. Additionally, references in retrieved articles were searched manually.

### Criteria for selection

**Figure 5:**
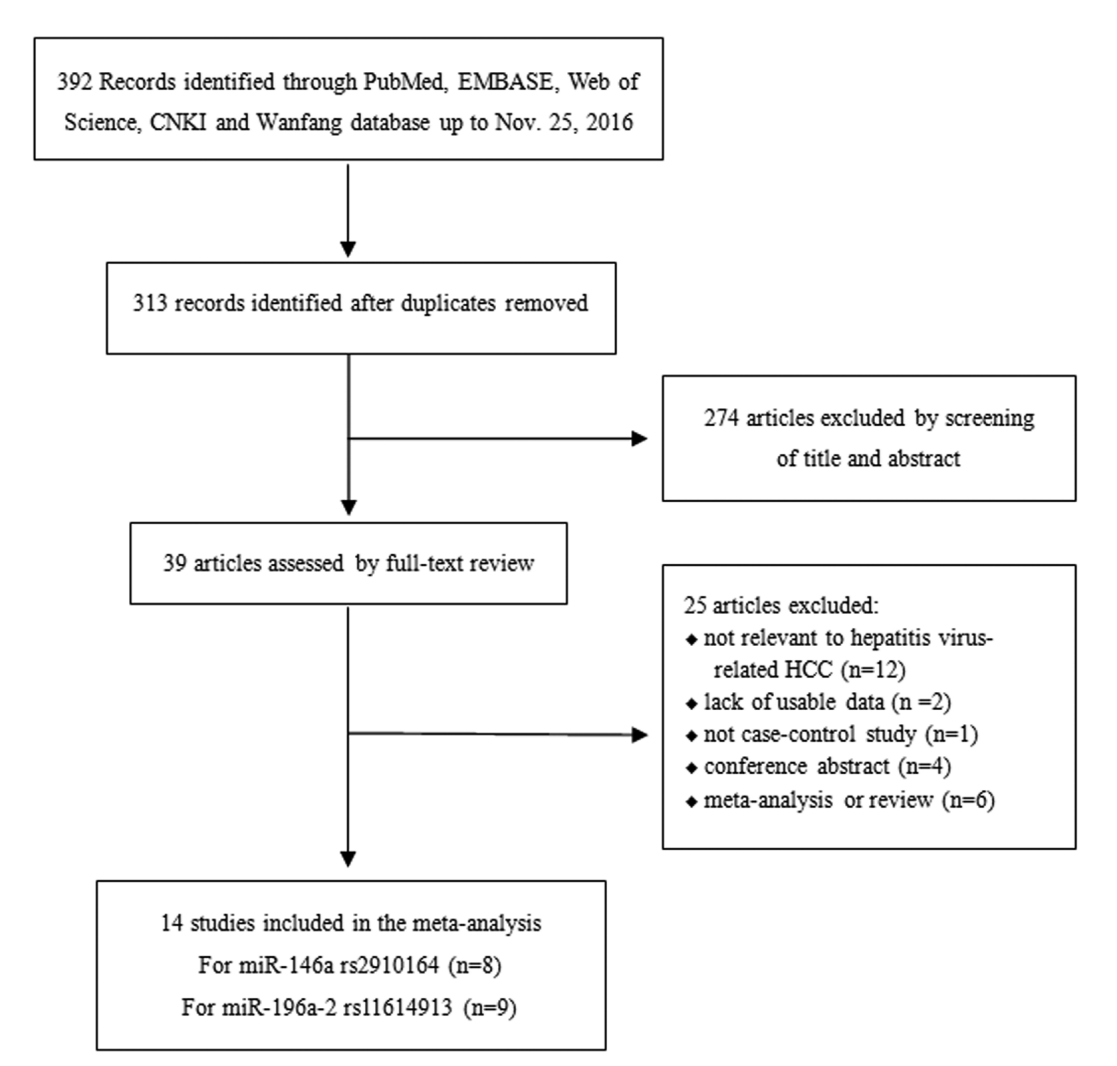
The flow chart illustrating the selection process of included studies.

The studies that complied with the following criteria were included: 1) original studies investigating the association of rs2910164 or rs11614913 and hepatitis virus-related HCC risk; 2) case-control design studies conducted in humans; 3) patients were pathologically diagnosed, with cancer-free individuals used as controls; 4) availability of full text, with detailed data of the allele and genotype distributions. Studies without a control group, repeated publications, reviews, and conference abstracts were excluded. If two or more studies showed an overlapping study population, only the most comprehensive study was included. The details of included and excluded articles were shown in Fig. 5.

### Data extraction

Based on the criteria listed above, 2 authors independently reviewed the literature. The quality of each included article was assessed by Newcastle-Ottawa Quality Assessment Scale for case-control studies. For each eligible study, the raw data and information including: first author, publication year, country of origin, ethnicity, genotype methods, source of control, numbers of cases and controls, frequencies of allele and genotypes, and *P* value of Hardy-Weinberg equilibriums (HWE) in controls were collected. Any discrepancy was discussed between authors to reach a consensus.

### Statistical analysis

Odds ratios (OR) with corresponding 95% confidence intervals (CI) for miR-146a and miR-196a-2 polymorphisms were calculated in each study under 5 different genetic models: allele comparison (B *vs*. A), homozygote (BB *vs*. AA), heterozygote (AB *vs*. AA), dominant model (BB+AB *vs*. AA), and recessive model (BB *vs.* AA+AB). The combined ORs were determined by Z test and *P*<0.05 was judged as statistically significant. Heterogeneity between studies was detected using I^2^ test and Q statistic, while significance was considered at I^2^>50%. A random-effects model was applied to analyze the pooled ORs if I^2^≥50%. Otherwise, a fixed-effects model was implemented. We carried out subgroup analyses to investigate the specific effects of ethnicity, source of control and etiology. Additionally, we assessed publication bias using Egger's test and Begg's funnel plot (significant bias was considered if *P*<0.05). We also performed a sensitivity analysis to assess the consistency and stability of our meta-analysis by removing each study in turn. All statistical analyses were accomplished with the software Review Manager 5.3 (Cochrane Collaboration, London, UK).and STATA (Version 12.0; Stata Corp, College Station, TX).

## SUPPLEMENTAL MATERIAL TABLES AND FIGURES



## Abbreviations

HCC: hepatocellular carcinoma
HBV: hepatitis B virus
HCV: hepatitis C virus
miRNA: microRNA
SNP: Single nucleotide polymorphism
OR: odds ratio
CI: confidence interval
HWE: Hardy-Weinberg equilibrium.
