# Supplementary material for: MiR-146a and miR-196a-2 polymorphisms are associated with hepatitis virus-related hepatocellular cancer risk: a meta-analysis
Source: Aging (Albany NY). 2017 Jan 31;9(2):381–90. doi: 10.18632/aging.101160 (PMC5361670; doi:10.18632/aging.101160)
Supplement: Supplementary file 1 [file aging-09-381-s001.pdf]

SUPPLEMENTARY MATERIAL

Table S1. Genotype distributions and allele frequencies of miR-146a and miR-196a-2 polymorphisms in cases and controls.

Study	Genotype (N)						Allele Frequency (N)				*P* of HWE
	Case			Control			Case		Control		
miR-146a rs2910164	CC	CG	GG	CC	CG	GG	C	G	C	G	
Zhou 2014	49	107	28	97	154	30	205	163	328	214	0.02
Cong 2014	46	39	19	117	84	17	131	77	318	118	0.84
Zhang 2013	257	390	124	367	475	156	904	638	1209	787	0.91
Shan 2013	33	25	13	78	71	36	91	51	227	143	0.08
Xiang 2012	21	34	18	33	46	21	98	120	112	88	0.5
Kim 2012	43	71	13	74	103	24	157	97	251	151	0.19
Wang 2011	58	103	38	138	185	61	219	179	461	307	0.94
Akkız 2011	8	67	113	11	67	144	83	293	89	355	0.4
miR-196a-2 rs11614913	CC	CT	TT	CC	CT	TT	C	T	C	T	
Yan 2015	63	113	51	88	136	63	239	215	312	262	0.41
Zhou 2014	67	97	20	66	160	55	231	137	292	270	0.06
Kou 2014	72	111	25	125	304	103	255	161	554	510	0.004
Hao 2013	58	90	21	67	160	55	206	132	294	270	0.07
Zhang 2013	171	376	224	165	502	328	718	823	832	1158	0.24
Han 2013	207	505	305	220	485	304	919	1115	925	1093	0.31
Kim 2012	24	70	33	45	107	49	118	136	197	205	0.36
Akkız 2011	64	70	19	58	87	40	198	108	203	167	0.49
Qi 2010	82	179	100	92	197	102	343	379	381	401	0.88

HWE: Hardy-Weinberg equilibrium

Table S2. Egger's test result of the two SNPs and hepatitis virus-related HCC risk based on allele frequency.

SNP	Std. Eff.	Coefficient	Std. Err.	t	*P*	95% CI
miR-146a C>G (rs2910164)	Slope	0.05	0.16	0.32	0.76	-0.34-0.44
	Bias	0.63	1.23	0.51	**0.63**	-2.39-3.64
miR-196a-2 C>T (rs11614913)	Slope	0.07	0.19	0.38	0.72	-0.38-0.52
	Bias	-2.05	1.88	-1.09	**0.31**	-6.50-2.41

Std. Eff.: standardized effect; Std. Err.: standard error; CI: confidence interval

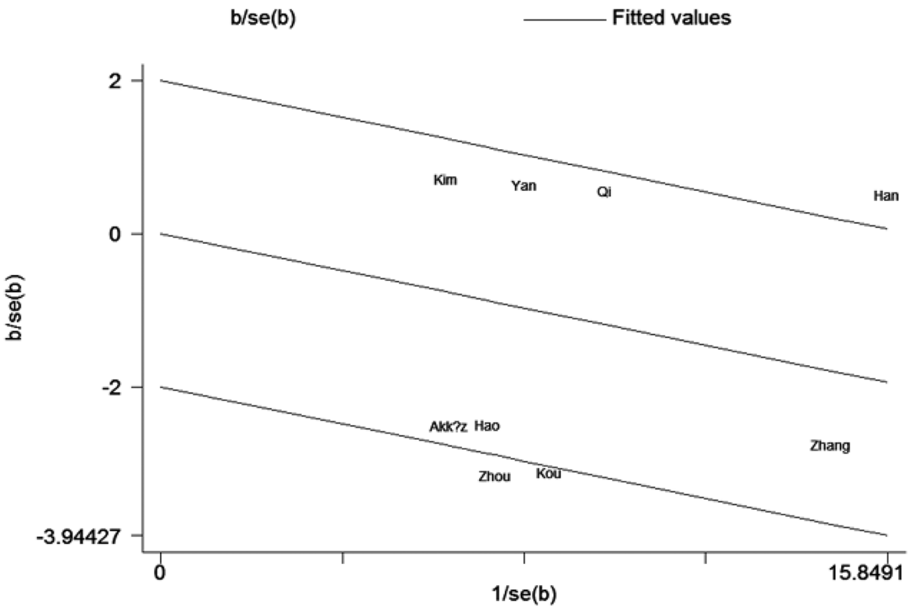

Supplementary Figure S1. Galbraith plots of miR-196a-2 rs11614913 and hepatitis virus-related HCC risk (under T *vs*. C).
